# MeCP2 inhibits ischemic neuronal injury by enhancing methylation of the FOXO3a promoter to repress the SPRY2-ZEB1 axis

**DOI:** 10.1038/s12276-022-00790-4

**Published:** 2022-08-01

**Authors:** Lei Meng, Bin Feng, Liming Luan, Zhihao Fang, Guangyu Zhao

**Affiliations:** Department of Neurosurgery, Shandong Provincial Hospital Affiliated to Shandong First Medical University, Jinan, 250021 P. R. China

**Keywords:** Neuroscience, Diseases

## Abstract

Methyl CpG binding protein 2 (MeCP2) is involved in nerve regeneration following ischemic stroke, but the related mechanism remains unclear. Here, we found low MeCP2 expression in hippocampal tissues. Using functional analysis, we demonstrated that MeCP2 accelerated FOXO3a methylation and subsequently inhibited its expression, thus repressing the apoptosis of neuronal cells. Mechanistically, FOXO3a could bind to the promoter region of SPRY2, consequently inducing its transcription and promoting the expression of the downstream target gene ZEB1. Altogether, our study revealed that overexpression of MeCP2 can protect mice against ischemic brain injury *via* disruption of the FOXO3a/SPRY2/ZEB1 signaling axis. Our results identify ectopic expression of MeCP2 as a therapeutic target in ischemic stroke.

## Stroke: Preventing nerve cell damage

The finding that an essential DNA-binding protein is present at reduced levels in the nerve cells of mice that have suffered ischemic strokes (IS), strokes caused by blockages in the blood supply to brain tissues, suggests new possibilities for treatment. Guangyu Zhao and colleagues at Shandong First Medical University in Jinan, China, propose that finding ways to stimulate increased production of the protein, called MeCP2, might help protect against and repair the nerve cell damage associated with IS. The authors suggest that MECP2’s nerve-regenerating effects involve enhanced addition of methyl groups (CH_3_) to a specific gene-regulating site on DNA. This represses a well-known molecular signaling pathway called the FOXO3a/SPRY2/ZEB1 axis. Further investigating these mechanisms and learning how to influence them should help clarify the possible clinical significance of these findings.

## Introduction

Ischemic stroke is a leading cause of death and disability globally^1^. Ischemic brain injury as a result of ischemic stroke has been well documented as a primary cause of neurological disability and diseases, including cognitive deficits, mental retardation and even persistent vegetative states^2^. Although extensive studies have focused on the mechanism of ischemic brain injury^3,4^, relatively few clinical therapeutics are available for ischemic brain injury at present^5^. However, greater efforts are required from an investigational standpoint to discover alternative therapeutic targets for ischemic stroke.

Noncoding RNAs^6^, such as microRNAs (miRNAs or miRs),^7^ or phytoestrogen^8^ have been reported in previous literature to exert neuroprotective effects against ischemic brain injury by regulating specific signaling pathways. Methylated CpG binding protein 2 (MeCP2) is an epigenetic factor that specifically recognizes and binds to DNA in the nucleus and is highly expressed in neuronal cells^9^. MeCP2 is located on the X chromosome, and its mutation is a key factor in initiating neurodevelopmental disorders, including cerebral ischemia^10,11^. The increased extent of MeCP2 phosphorylation driven by repetitive transcranial magnetic stimulation contributes to the improvement of neurological functions and reduction of the area of cerebral infarction^12^. Additionally, MeCP2 is involved in the regulation of neuralgia mediated by the superficial dorsal horn and dorsal root ganglia^13^. As previously reported, MeCP2 can affect the functionality of cells by methylating the Forkhead box O 3a (FOXO3a) promoter region to regulate its transcription, ultimately impacting autophagy^14^. The transcription factor FOXO3a is considered a key regulator of cellular homeostasis, stress response and longevity due to its regulatory roles in a variety of stress responses following oxidative stress, nutrient shortage, heat shock, hypoxia and DNA damage^15^. Extensive studies have demonstrated the involvement of FOXO3a in various pathways, providing neuroprotection against ischemic brain injury. For example, a previous report implicated the JNK/FOXO3a/Bim pathway in the regulation of neuronal apoptosis and infarct volume in the brain tissues of rats subjected to hypoxia-ischemia^16^. Additionally, mild hypothermia pretreatment was shown to protect the liver against ischemia and reperfusion injury *via* the PI3K/Akt/FOXO3a pathway^17^. More importantly, SPROUTY2 (SPRY2), which is involved in cell growth, differentiation and tumorigenesis, is a putative target gene of FOXO3a^18^, and the downregulation of SPRY2 expression mediated by small interfering RNA (siRNA) was reported to diminish ischemic brain injury^19^. Moreover, genetic reduction of SPRY2 in mice was shown to be neuroprotective by stimulating ischemic brain injury-induced astrogliosis that is capable of limiting neuronal cell death and lesion size^19^. Thus, we hypothesized that MeCP2 could improve neuronal injury in ischemic brain injury via the interaction of FOXO3a and SPRY2.

## Materials and methods

### Ethics statement

The study was ratified by the Ethics Committee of Experimental Animal Care and Use of Shandong Provincial Hospital Affiliated to Shandong First Medical University (approval number: 201903022) and strictly performed according to the Guide for the Care and Use of Laboratory Animals published by the US National Institutes of Health.

### Mouse model construction

Sixty male C57BL/6 J mice weighing 22 to 25 g (8–10 weeks old) were provided by Beijing HFK Bioscience Cooperation (Beijing, China). The mice were housed under controlled conditions of 22 °C and 70% humidity with a 12 h light/dark cycle. The mice were granted free access to food and water. The mice were subjected to middle cerebral artery occlusion (MCAO)^20^. Sham-operated mice underwent the same surgical procedure, with the exception of MCAO. After MCAO, the mice were subjected to anesthesia with 5% isoflurane and cervical dislocation, followed by collection of the brain. Regional cerebral blood flow (rCBF) before ischemia was monitored to guarantee the rigor of the MCAO experiments. During MCAO, a Laser Speckle PeriCam PSI System (Perimed AB, Jarfalla, Sweden) was employed for monitoring purposes. Mice were excluded from the study and euthanized when rCBF was not reduced to at least 25% of the initial level. Lentiviral vectors with oe-MeCP2 and oe-FOXO3a were constructed using the lentiviral overexpression vector LV5-green fluorescent protein (GFP, VL3211, Shanghai GenePharma Co., Ltd., Shanghai, China).

Lentiviruses expressing overexpressed MeCP2 and FOXO3a and their empty vectors were utilized to infect mouse brain tissues. Sixty mice were divided into sham-operated and MCAO-treated mice, and the MCAO-treated mice were further infected with lentiviruses expressing overexpression negative control (oe-NC), oe-MeCP2, oe-MeCP2 + oe-NC and oe-MeCP2 + oe-FOXO3a (*n* = 10 for mice upon each treatment). Lentiviruses (5 µL of 10^8^ TU/mL; GenePharma, Shanghai, China) were stereotaxically injected into the lateral ventricle of mice with a Hamilton microsyringe (Hamilton Co., Reno, NV, USA) 2 weeks before MCAO.

### Primary hippocampal neuronal cell model establishment

One-day-old mice were externalized under 5% isoflurane anesthesia. The hippocampus was then dissected and trypsinized. The cells were subsequently isolated by trituration 10 times using 3 different sizes of fire-polished Pasteur pipettes and cultured in Dulbecco’s modified Eagle’s medium (DMEM) (Gibco, Grand Island, NY, USA) with 10% fetal bovine serum (FBS). The cells were filtered utilizing a 40 mm filter and seeded in 8-well poly-L-lysine-treated chamber slides at a density of 30,000 cells per well, after which they were cultured in DMEM containing 4.5 g/L glucose, 100 U/mL penicillin, 100 μg/mL streptomycin, 2 mM glutamine and B27 at 37 °C under 5% CO_2_.

The establishment of the oxygen-glucose deprivation (OGD)-induced cell model was conducted based on a previously reported method^7^. Normal neuronal cells without OGD induction were regarded as the control.

### Cell transfection and grouping

A lentivirus packaging system was constructed utilizing LV5-GFP (lentiviral overexpression vectors) and pSIH1-H1-copGFP (lentiviral short hairpin RNA [shRNA] vectors). shRNA targeting FOXO3a shRNA, SPRY2 shRNA, and NC (sh-NC) was constructed by Shanghai GenePharma Co., Ltd. (Shanghai, China). HEK293T cells were cotransfected with the packaging virus as well as the target vector, and the supernatant was collected after a 48 h period of cell culture. After centrifugation, the virus particles in the supernatant were filtered, and the virus titer was determined. The virus at the exponential growth phase was collected. OGD-treated cells were then infected with lentiviruses expressing oe-NC, oe-MeCP2, oe-FOXO3a, sh-NC, sh-SPRY2, oe-FOXO3a + sh-NC, oe-FOXO3a + sh-SPRY2, oe-MeCP2 + oe-NC and oe-MeCP2 + oe-FOXO3a.

### TTC (2,3,5-triphenyltetrazolium chloride) staining

The mice were euthanized at 24 h following MCAO. Their brains were then collected and cut into 2-mm-thick sections, which were stained with 2% TTC (Sigma-Aldrich, St. Louis, MO, USA) at 37 °C for 10 min. The infarct volume (%) was assessed by ImageJ software (Bethesda, MD, USA).

### Behavior test

The modified neurologic severity score (mNSS), rotarod test and grip strength test were performed to assess the neurological deficits of the mice post-MCAO^21^.

### Reverse transcription quantitative polymerase chain reaction (RT-qPCR)

Total RNA was extracted from cells or tissue samples using TRIzol reagent (Invitrogen, Thermo Fisher, USA). A NanoDrop ND-1000 ultraviolet-visible spectrophotometer (Nanodrop Technologies, Wilmington, DE, USA) was used to determine both the quality and the concentration of the extracted RNA. Total RNA was extracted utilizing the RNeasy Mini Kit (Qiagen, Valencia, CA, USA), while mRNA was detected utilizing a reverse transcription kit (RR047A, TaKaRa, Japan) to obtain complementary DNA (cDNA). Subsequently, the cDNA was applied as a template, after which RT-qPCR was performed with a SYBR^®^ Premix Ex Taq^TM^ II (Perfect Real Time) kit (DRR081, TaKaRa, Japan) utilizing an ABI 7500 instrument (Applied Biosystems, Foster City, CA, USA). Glyceraldehyde 3-phosphate dehydrogenase (GAPDH) was employed as the internal control for mRNA levels to normalize the results. The primer sequences are depicted in Supplementary Table 1. The fold changes were calculated using relative quantification (the 2^−△△Ct^ method).

### Western blot analysis

Total protein was extracted, and its concentration was determined by a bicinchoninic acid assay kit (Beyotime, Shanghai, China). After 10% sodium dodecyl sulfate-polyacrylamide gel electrophoresis separation, the protein was transferred to a polyvinylidene fluoride membrane (Millipore, Billerica, MA, USA) and blocked with 5% skim milk powder for 1 h. Incubation was then performed at 4 °C overnight with primary antibodies (Abcam, Inc., Cambridge, UK): mouse monoclonal antibody to MeCP2 (ab50005, 1:1000), rabbit polyclonal antibody to SPRY2 (ab85670, 1:1000), rabbit polyclonal antibody to FOXO3A (ab23683, 1:1000), rabbit monoclonal antibody to Bax (ab32503, 1:1000), rabbit monoclonal antibody to Bcl-2 (ab182858, 1:1000) and rabbit monoclonal antibody to cleaved caspase-3 (ab214430, 1:5000) as well as with horseradish peroxidase (HRP)-labeled secondary goat-anti-rabbit (TransGen Biotech) at room temperature for 1 h. The immunoblots were then visualized utilizing enhanced chemiluminescence (JK30026.3, Shanghai Baoman Biotechnology Co., Ltd., Shanghai, China), and the band intensities were quantified utilizing ImageJ software. Rabbit polyclonal antibody against GAPDH (ab37168, 1:1000, Rabbit, Abcam, Inc., Cambridge, UK) was used as an internal reference.

### Terminal deoxynucleotidyl transferase-mediated dUTP-biotin nick end labeling (TUNEL) assay

The hippocampal tissue sections of the mice from each group were submitted to TUNEL staining according to the TUNEL kit instructions (Roche, USA). Briefly, the sections were heated at 60 °C for 15 min, dewaxed with xylene, hydrated with gradient alcohol, and digested with Proteinase K followed by 3 PBS washes. TUNEL solution was added to the sections and incubated at 37 °C for 1 h. Next, 0.3% H_2_O_2_ methanol was added to the sections at room temperature for 10 min, followed by 3 PBS washes. Then, streptavidin-biotin peroxidase complex was added and incubated for 30 min at 37 °C, followed by 3 PBS washes. The sections were stained with diaminobenzidine, counterstained with hematoxylin, dehydrated by gradient ethanol, cleared with xylene, and mounted with neutral balata. The sections were observed under a light microscope, and the apoptosis index was subsequently calculated.

### Immunofluorescence assay

The hippocampal tissues of the mice from different groups were treated with normal saline, fixed with 4% paraformaldehyde for 24 h, treated with 0.1 M PBS and subsequently dehydrated in graded ethanol after being embedded in paraffin. An immunofluorescence assay was then performed on 3.5 μm-thick coronal sections. Following retrieval, the sections were incubated in 0.1 M PBS containing 10% serum for 40 min and incubated overnight at 4 °C with primary antibodies: mouse monoclonal antibody [Mec-168] against MeCP2 (ab50005, 1:1000, Abcam, Inc., Cambridge, UK) and rabbit polyclonal antibodies against SPRY2 (ab85670, 1:1000, Abcam, Inc., Cambridge, UK) and NeuN (mouse 1:1,000; 2,742,283, Millipore, Billerica, MA, USA). After three 0.1 M PBS washes (5 min per wash), the sections were incubated with fluorescein isothiocyanate (FITC)-conjugated secondary goat anti-rabbit immunoglobulin G (IgG) (1:100; SA00003-2, Proteintech Group, Chicago, IL, USA) or tetramethylrhodamine isothiocyanate-conjugated anti-mouse IgG (1:100; SA00007-1, Proteintech Group, Chicago, IL, USA) at room temperature for 4 h. The sections were counterstained with 4’,6-diamidino-2-phenylindole for 5 min. Immunofluorescence images were obtained using a Nikon Eclipse NI microscope.

### Cell counting Kit-8 (CCK-8) assay

A CCK-8 assay (Beyotime, Shanghai, China) was used to measure the viability of neurons. Cells were then seeded in 96-well plates at a density of 1 × 10^4^ cells/well. After 24 h, 10 μL of CCK-8 solution was added to the cells for 1–2 h with the reaction taking place at 37 °C, followed by determination of the optical density (OD) value at 450 nm.

### Bisulfite sequencing PCR (BSP) assay

Bisulfite sequencing primers were designed, and the primer sequences are shown in Supplementary Table 2. The PCR steps and conditions were the same as those of methylation-specific PCR (MSP). The PCR product was separated via agarose gel electrophoresis. The target band was cut under UV irradiation and placed in a 1.5 mL EP tube. DNA was recovered using an agarose gel DNA recovery kit (Tiangen Biotechnology Co., Ltd., Beijing, China). The clone reaction system was set as follows: 5.5 μL of PCR products amplified by bisulfite sequencing primers, 1 μL of T-easy, 1 μL of ligase, and 7.5 μL of 2 × buffer (pGEM-T Easy Vector Systems, Promega Corp., Madison, Wisconsin). The clone reaction mixture was incubated at 4 °C overnight. Next, 10 μL of the ligation products was added to 100 μL of DH5α competent cells, mixed by means of gentle flicking, placed on ice for 30 min, followed by heat shock at 42 °C for 45 s, and incubated on ice for 1 min. The mixture was then added to 800 μL of SOC liquid medium and cultured by shaking at 37 °C for 60 min. The mixture was cultured in LB solid medium containing X-gar, IPTG and ampicillin in a 37 °C incubator for amplification. At least ten individual colonies (white colonies) were selected, and the BcaBEST sequencing primers and MS universal primers were utilized for colony PCR. Positive recombinants were screened by agarose gel electrophoresis and cultured overnight for subsequent sequencing.

### MSP assay

Genomic DNA extraction was performed with a genomic DNA purification kit (Qiagen, Hilden, Germany). Bisulfite modification of DNA was performed with an Intergen CpGenome DNA Modification Kit (Intergen Company, New York, USA). The unmethylated cytosine was converted to uracil using bisulfite, while the methylated cytosine remained intact. MSP was implemented with FOXO3a methylation-specific primers (Supplementary Table 2). The total volume of the amplification reaction system was 25 μL, which consisted of 12.5 μL of Hot-StarTaq Master Mix (Qiagen, Hilden, Germany), 1 μL of bisulfite-treated DNA template, and 1.5 μL of forward and reverse primers. The PCR products were analyzed on a 3% TBE agarose gel and then visualized and analyzed using a gel imaging system.

### Chromatin immunoprecipitation (ChIP) assay

A ChIP assay kit (cat# 26156, Thermo Fisher Scientific, USA) was employed with rabbit polyclonal antibody against MeCP2 (ChIP Grade) (ab2828, Abcam, Inc., Cambridge, UK), mouse monoclonal antibody against histone H3K9me2 (ChIP Grade) (ab1220, Abcam, Inc., Cambridge, UK), and rabbit polyclonal antibody against FOXO3a (ChIP Grade) (ab12162, Abcam, Inc., Cambridge, UK). The primers for the FOXO3a promoter were as follows: forward, 5′-CAAACCTTTTGGTGCCTGAT-3′ and reverse, 5′-GTGTCCGGTTCCCTGTTAGA-3′. Primers for the SPRY2 promoter were as follows: forward, 5’-GTTTCCAGTCCTTCAAGCAATC-3’ and reverse, 5’-AATTGGGAGTGGCTGTAACAAA-3’.

### Dual luciferase reporter assay

The cells were transfected with X-tremeGENE HP DNA reagent (Roche). In transient transfection, firefly and Renilla luciferase activities were assessed with a Dual Luciferase Assay kit (Promega) and a Centro XS^3^ LB960 luminometer (Berthold, Bad Wildbad, Germany), respectively. The activity of the SPRY2 promoter was evaluated utilizing the pGL-hSPRY2-Luc (-2000) vector.

### Statistical analysis

Data analysis was performed with SPSS 21.0 software (IBM Corp. Armonk, NY, USA). Measurement data are presented as the mean ± standard deviation based on a minimum of three independent experiments. Comparisons between groups were performed with an unpaired *t* test, while comparisons between multiple groups were performed with one-way analysis of variance (ANOVA) with Tukey’s post-hoc test. Comparisons for different rotational speeds were performed with two-way ANOVA followed by Bonferroni post hoc test. Comparisons among multiple groups at different time points were performed with repeated-measures ANOVA with Bonferroni post hoc test. *p* < 0.05 was considered to indicate a significant difference.

## Results

### MeCP2 expression is downregulated in MCAO-treated mice and OGD-induced cell models

**Fig. 1:**
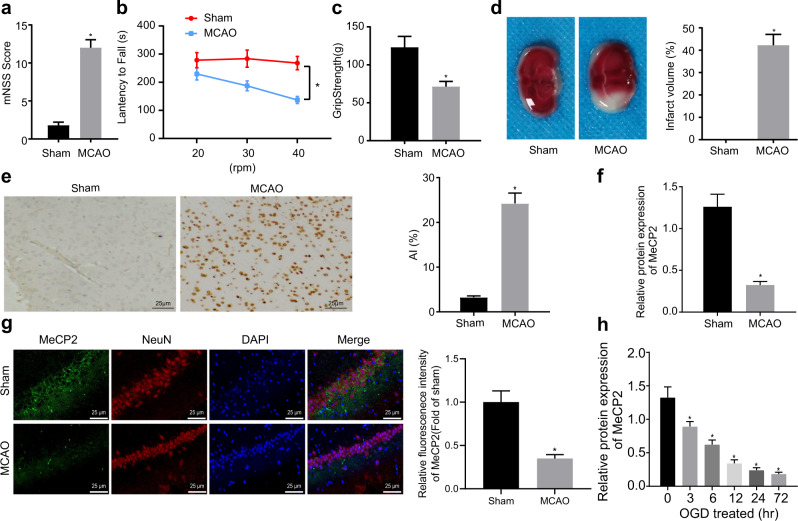
MeCP2 expression is downregulated in the MCAO-treated mice and OGD-treated cell models. **a** The mNSS of the sham-operated mice (*N* = 10) and the MCAO-treated mice (*N* = 10); **b** The latency period to fall from the rod of the sham-operated mice (*N* = 10) and the MCAO-treated mice (*N* = 10); **c** Grip strength of the sham-operated mice (*N* = 10) and the MCAO-treated mice (*N* = 10); **d** TTC staining of infarct volume in the sham-operated mice (*N* = 10) and the MCAO-treated mice (*N* = 10); **e** Cell apoptosis in the hippocampus of the sham-operated mice (*N* = 10) and the MCAO-treated mice (*N* = 10) detected by TUNEL staining (×400); **f** Western blot analysis of MeCP2 protein expression in hippocampal tissues of the sham-operated mice (*N* = 10) and the MCAO-treated mice (*N* = 10); **g** MeCP2 expression in hippocampal neurons of the sham-operated mice (*N* = 10) and the MCAO-treated mice (*N* = 10) examined by immunofluorescence (×400); **h** Western blot analysis of MeCP2 protein in the OGD-treated cells. In Panels A-G, data were compared by unpaired *t* tests. In Panel **h**, data were compared by one-way ANOVA with Tukey’s post-hoc test. *p < 0.05 vs. the sham-operated mice or cells treated with OGD at 0 h.

The behavior test showed that the MCAO-treated mice exhibited an increase in mNSS (Fig. 1a), while the latency to fall from the rod (Fig. 1b) and grip strength (Fig. 1c) were reduced. TTC staining revealed that the cerebral infarct volume in the MCAO-treated mice was increased (Fig. 1d). In addition, the TUNEL assay revealed that the hippocampal apoptosis rate was higher in the MCAO-treated mice (Fig. 1e), suggesting the successful construction of MCAO mouse models. Furthermore, MeCP2 expression was reduced in the hippocampal tissues of the MCAO-treated mice (Fig. 1f, Supplementary Fig. 1a). Immunofluorescence assays indicated that the expression of MeCP2 in the hippocampal neurons of the MCAO-treated mice was lower (Fig. 1g). Next, a progressive decrease in MeCP2 in hippocampal neurons over time with OGD treatment was found (Fig. 1h). Taken together, these results suggested that MeCP2 was expressed at low levels in ischemic brain injury.

### Overexpression of MeCP2 protects mice against ischemic brain injury

**Fig. 2:**
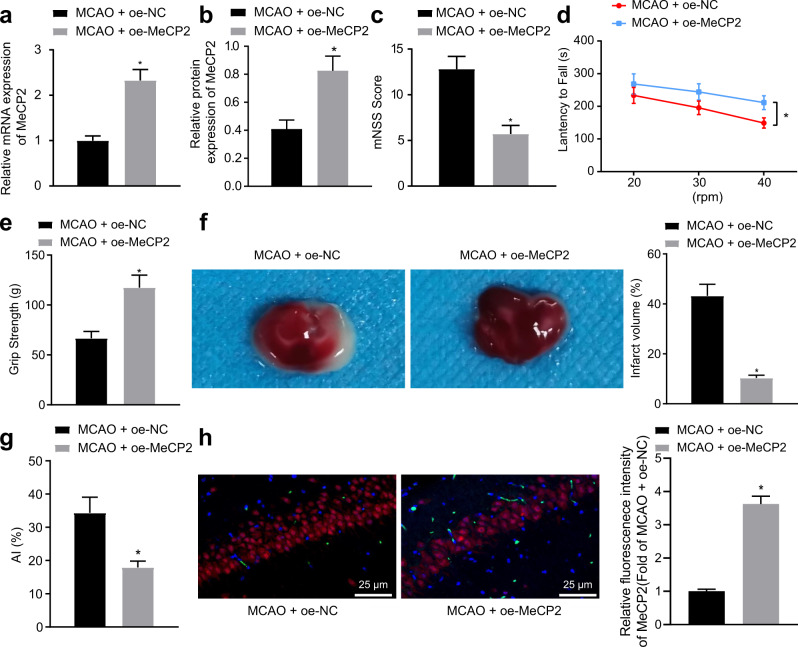
Overexpression of MeCP2 inhibits ischemic brain injury in the mouse MCAO model. **a** The mRNA expression of MeCP2 in the hippocampus of the MCAO + oe-MeCP2-treated mice quantified by RT-qPCR; **b** The protein expression of MeCP2 in the hippocampus of the MCAO + oe-MeCP2-treated mice measured by western blot analysis; **c** The mNSS test of the MCAO + oe-MeCP2-treated mice; **d** The rotarod test of the MCAO + oe-MeCP2-treated mice; **e** Grip strength test for evaluating neuroethology of the MCAO + oe-MeCP2-treated mice; **f** Infarct volume of the MCAO + oe-MeCP2-treated mice measured by TTC staining; **g** Cell apoptosis in the hippocampus of the MCAO + oe-MeCP2-treated mice characterized by TUNEL staining. **h** Expression of MeCP2 in hippocampal neurons of the oe-MeCP2-treated mice measured by immunofluorescence assays (×400). Data were compared by unpaired t tests. *p < 0.05 vs. the MCAO + oe*-*NC-treated mice. *N* = 10 for each treatment group.

We then intended to clarify the molecular mechanism of MeCP2 in ischemic injury in vivo. We found upregulated MeCP2 expression in the oe-MeCP2-treated mice (Fig. 2a, b, Supplementary Fig. 1b). The behavior test revealed that the mNSS of the oe-MeCP2-treated mice was decreased (Fig. 2c), while the latency period to fall from the rod was increased (Fig. 2d), in addition to increased grip strength (Fig. 2e). TTC staining indicated that the infarct volume was decreased in the oe-MeCP2-treated mice (Fig. 2f). Moreover, the cell apoptosis rate of the oe-MeCP2-treated mice was reduced (Fig. 2g). Furthermore, the expression of MeCP2 in hippocampal neurons of the oe-MeCP2-treated mice measured by immunofluorescence assays showed an increase (Fig. 2h). The aforementioned results indicated that overexpression of MeCP2 conferred protection against ischemic brain injury in mice.

### Overexpression of MeCP2 inhibits OGD-induced apoptosis of neuronal cells

**Fig. 3:**
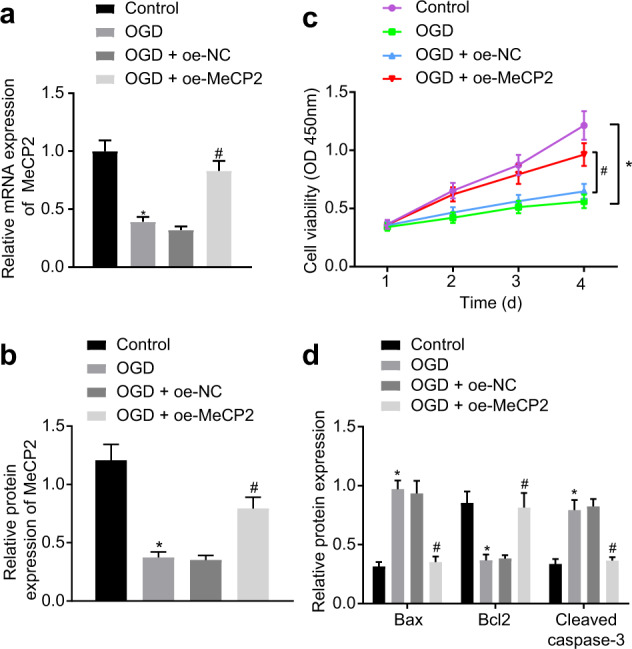
MeCP2 inhibits OGD-induced cell injury by repressing apoptosis of neuronal cells. **a**, **b** The mRNA expression of MeCP2 in the OGD-treated neuronal cells determined by RT-qPCR; **b** The protein expression of MeCP2 in the OGD-treated neuronal cells measured by western blot analysis; **c** The cell viability characterized by CCK-8 assays; **d** Western blot analysis of apoptotic proteins: Bax, Bcl2, and cleaved caspase-3 in cells. In Panels **a**, **b**, and **d**, the data were compared by ANOVA with Tukey’s post-hoc test. In Panel **c**, data were compared using repeated-measures ANOVA. *p < 0.05 vs. the untreated cells; ^#^*p* < 0.05 vs the OGD + oe-NC-treated cells.

To further investigate the role of MeCP2 in the apoptosis of ischemic neurons in vitro, we constructed OGD-deprived cell models. The expression of MeCP2 was reduced in the OGD-treated cells. When compared with that of the OGD + oe-NC-treated cells, the expression of MeCP2 was increased in the OGD + oe-MeCP2-treated cells (Fig. 3a, b). In addition, cell viability was reduced after OGD treatment and was rescued upon MeCP2 overexpression (Fig. 3c). Moreover, the protein expression of Bax and cleaved caspase-3 was upregulated in the OGD-treated cells, while that of Bcl-2 was downregulated. In the OGD-treated cells, the expression of Bax and cleaved caspase-3 was decreased; however, that of Bcl-2 was increased upon MeCP2 overexpression (Fig. 3d). In summary, the above results indicated that MeCP2 inhibited OGD-induced neuronal cell injury by suppressing apoptosis.

### MeCP2 inhibits FOXO3a transcription by methylating its promoter region

**Fig. 4:**
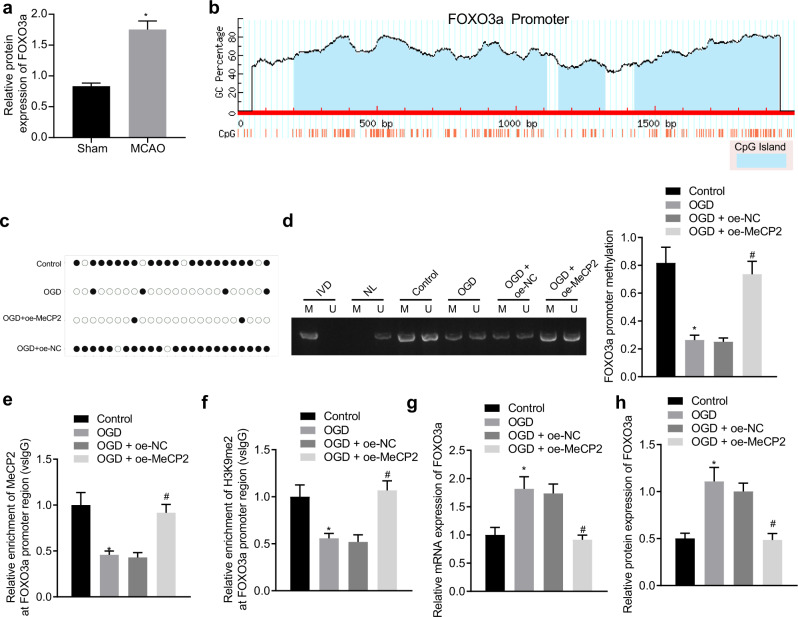
MeCP2 methylates the promoter region of FOXO3a and suppresses its expression. **a** Western blot analysis of FOXO3a expression in the hippocampus of the sham-operated or MCAO-treated mice normalized to GAPDH; **b** CpG islands of the FOXO3a promoter region predicted by the Methprimer website; **c** Methylation status of the FOXO3a promoter region after overexpression of MeCP2 in hippocampal neuronal cells measured by BSP experiments; **d** Methylation status of the FOXO3a promoter region after overexpression of MeCP2 in cells detected by MSP experiments. (IVD: in vitro methylated DNA, positive control; NL: unmethylated positive control; U: unmethylation; M: methylation); **e** Enrichment of MeCP2 in the FOXO3a promoter region characterized by ChIP experiments; **f** Enrichment of H3K9me2 in the FOXO3a promoter region displayed by ChIP experiments; **g** The mRNA expression of FOXO3a in neuronal cells with MeCP2 overexpression quantified by RT-qPCR; **h** The protein expression of FOXO3a in neuronal cells with MeCP2 overexpression measured by western blot analysis; Data were compared by unpaired *t* tests. *p < 0.05 vs. the sham-operated mice or control cells.

To explore how MeCP2 suppresses FOXO3a transcription, we performed western blot analysis and verified that FOXO3a was significantly overexpressed in the MCAO-treated mice compared with sham-operated mice (Fig. 4a, Supplementary Fig. 1c). The Methprimer website provided an indication of the existence of CpG islands in the FOXO3a promoter region (Fig. 4b). Thus, we subsequently hypothesized that MeCP2 could regulate ischemic injury through methylation of FOXO3a. We subsequently examined the methylation status of the FOXO3a promoter region after overexpression of MeCP2 in hippocampal neuron cells by BSP and MSP experiments. The methylation level of the FOXO3a promoter region following OGD induction was significantly decreased, while oe-MeCP2 treatment caused an increase (Fig. 4c, d). The ChIP assay demonstrated that the enrichment of MeCP2 in the FOXO3a promoter region following OGD induction was significantly decreased, while the opposite trend was detected after oe-MeCP2 treatment (Fig. 4e). In addition, the enrichment of H3K9me2 in the FOXO3a promoter region was significantly decreased in the OGD group, while the opposite finding was observed following oe-MeCP2 treatment (Fig. 4f). We also found an enhancement in the mRNA and protein levels of FOXO3a following OGD treatment, while they were reduced after oe-MeCP2 treatment (Fig. 4g, h, Supplementary Fig. 1d). Taken together, the data obtained indicated that MeCP2 was recruited to the promoter region of FOXO3a to augment its methylation and H3K9 dimethylation, ultimately inhibiting the expression of FOXO3a.

### MeCP2 protects neurons against OGD-induced ischemic injury *via* the FOXO3a gene

**Fig. 5:**
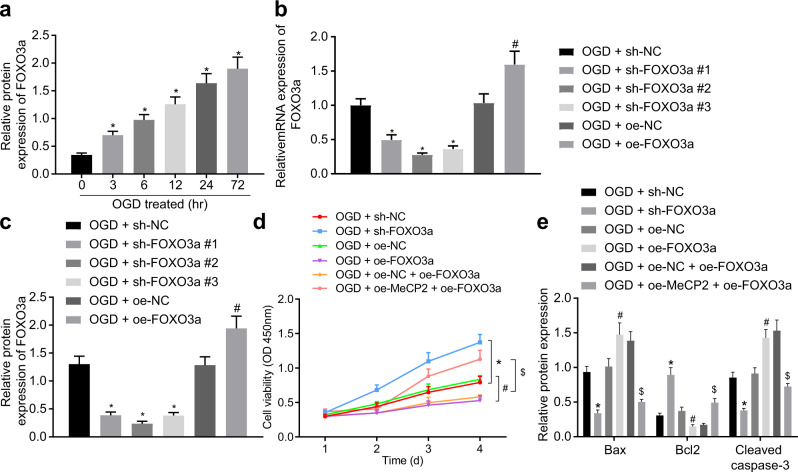
MeCP2 protects neuronal cells against OGD-induced injury by suppressing the expression of FOXO3a. **a** Western blot analysis of FOXO3a protein in cells; **b** The mRNA expression of FOXO3a in cells detected by RT-qPCR; **c** The protein expression of FOXO3a in cells detected by western blot analysis; **d** Cell viability detected by CCK-8 assays; **e** Western blot analysis of the apoptosis-related proteins Bax, Bcl-2 and cleaved caspase-3 in cells. In Panels **a**, **b**, **c**, and **e**, the data were compared by one-way ANOVA with Tukey’s post-hoc test. In Panel **d**, data were compared by repeated-measures ANOVA with a Bonferroni post hoc test. *p < 0.05 *vs*. the cells treated with OGD at 0 h or OGD + sh-NC-treated cells; ^#^*p* < 0.05 vs. the OGD + oe-NC-treated cells; ^$^*p* < 0.05 vs. the OGD + oe-NC + oe-FOXO3a-treated cells.

To further investigate the effect of downregulated FOXO3a expression on ischemic injury of neuronal cells, we performed western blot analysis, and the results showed that the expression of FOXO3a was increased with OGD treatment time (Fig. 5a), highlighting the involvement of FOXO3a in OGD-induced neuronal cell injury. Next, the OGD-exposed neuronal cells were infected with lentiviruses expressing sh-FOXO3a and oe-FOXO3a, and the efficiency was determined using RT-qPCR and western blot analysis. In the OGD-exposed neuronal cells, the expression of FOXO3a was successfully reduced by lentiviruses expressing sh-FOXO3a#1, sh-FOXO3a#2, and sh-FOXO3a#3, in which sh-FOXO3a#2 with the best knockdown efficiency was chosen for the subsequent experiments. In addition, FOXO3a was successfully overexpressed in the OGD-exposed neuronal cells by lentivirus expressing oe-FOXO3a (Fig. 5b, c). The CCK-8 assay demonstrated that the viability of the OGD-exposed neuronal cells was enhanced when FOXO3a was knocked down, while it was impaired in response to FOXO3a overexpression (Fig. 5d). Western blot analysis revealed that the expression of Bax and cleaved caspase-3 was reduced, while the expression of Bcl-2 was increased in the OGD-exposed neuronal cells after FOXO3a silencing, in contrast to the changes caused by overexpression of FOXO3a (Fig. 5e). To further investigate whether FOXO3a-mediated cell apoptosis could be modulated by MeCP2, we performed rescue experiments in the OGD-exposed neuronal cells. Enhancement of MeCP2 diminished the viability of the OGD-exposed neuronal cells overexpressing FOXO3a (Fig. 5d). Additionally, MeCP2 overexpression reversed the effects of FOXO3a overexpression on the protein expression of Bax, cleaved caspase 3, and Bcl-2 (Fig. 5e). In summary, the experimental data supported the hypothesis that MeCP2 protected neuronal cells against OGD-induced neuronal cell injury by suppressing FOXO3a.

### FOXO3a binds to the SPRY2 promoter to augment its transcription

**Fig. 6:**
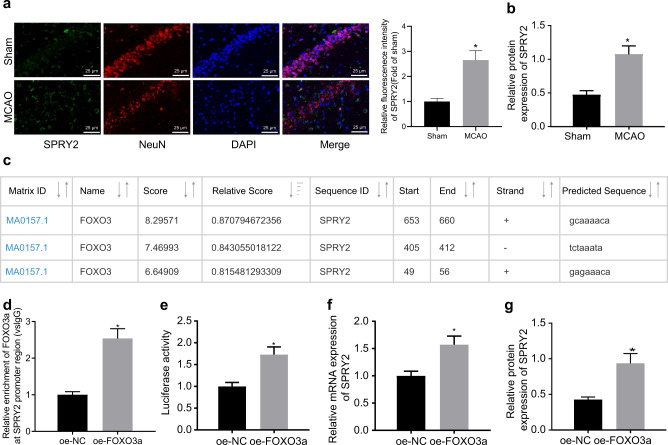
FOXO3a is recruited to the SPRY2 promoter region to augment its transcription and expression of downstream target genes in ischemic neurons. **a** The expression of SPRY2 in hippocampal neuronal cells examined by immunofluorescence (×400); **b** The expression of SPRY2 in hippocampal tissues examined by western blot analysis; **c** Binding sites between FOXO3a and the SPRY2 promoter region predicted by the JASPAR website; **d** The enrichment of FOXO3a in the SPRY2 promoter region analyzed by ChIP; **e** The effect of FOXO3a on the activity of the SPRY2 promoter characterized by dual luciferase experiments; **f** The mRNA expression of SPRY2 in the oe-FOXO3a-treated neuronal cells quantified by RT-qPCR. **g** The protein expression of SPRY2 in the oe-FOXO3a-treated neuronal cells quantified by western blot analysis. In Panels **b**, **d** and **e**–**g**, data were compared by unpaired t tests. In Panel a, data were compared by one-way ANOVA with a Tukey’s post-hoc test. *p < 0.05 vs. the sham-operated mice or oe-NC-treated cells.

To test the hypothesis that FOXO3a regulates ischemic brain injury through SPRY2, we initially examined the expression of SPRY2 in hippocampal neuronal cells using immunofluorescence, the result of which illustrated that SPRY2 was highly expressed in hippocampal neuronal cells of the MCAO-operated mice compared to the sham-operated mice (Fig. 6a). The high expression of SPRY2 in hippocampal tissues was also confirmed by western blot analysis (Fig. 6b, Supplementary Fig. 1e). The JASPAR website predicted the existence of a binding site between FOXO3a and the SPRY2 promoter region (Fig. 6c). The ChIP results revealed that the enrichment of FOXO3a in the SPRY2 promoter region was increased in the oe-FOXO3a-treated cells (Fig. 6d). The effect of FOXO3a on the activity of the SPRY2 promoter was subsequently detected by dual-luciferase reporter assays, and the results demonstrated that the luciferase activity of the SPRY2 promoter was enhanced by oe-FOXO3a (Fig. 6e). The expression of SPRY2 was increased following FOXO3a overexpression (Fig. 6f, g, Supplementary Fig. 1f). Taken together, the data displayed here provide evidence that FOXO3a is recruited to the promoter region of SPRY2 to augment its transcription in ischemic neurons.

### FOXO3a facilitates ischemic neuronal injury by promoting the transcription of SPRY2

**Fig. 7:**
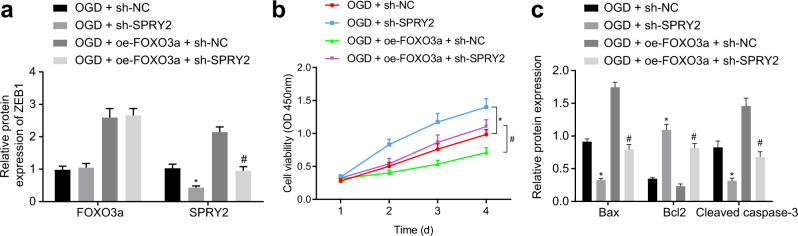
FOXO3a facilitates ischemic neuronal injury in the hippocampus by stimulating SPRY2 transcription. **a** The expression of FOXO3a and SPRY2 in cells analyzed by western blot analysis; **b** The cell viability measured by CCK-8 assays; **c** The expression levels of apoptosis-related proteins, Bax, Bcl2 and cleaved caspase-3, examined by western blot analysis. In Panels **a** and **c**, data were compared by one-way ANOVA with Tukey’s post-hoc test. In Panel **b**, data were compared by repeated-measures ANOVA with a Bonferroni post hoc test. *p < 0.05 *vs*. the OGD + sh-NC-treated cells; ^#^*p* < 0.05 vs. the OGD + oe-FOXO3a + sh-NC-treated cells.

Here, we attempted to exploit the effect of FOXO3a on ischemic neuronal injury. We observed that compared with that in the ODG + sh-NC-treated cells, the expression of SPRY2 was markedly reduced in the OGD + sh-SPRY2-treated cells, and compared with that in the OGD + oe-FOXO3a + sh-NC-treated cells, the expression of SPRY2 was significantly decreased in the OGD + oe-FOXO3a + sh-SPRY2-treated cells (Fig. 7a). Cell viability was increased after SPRY2 knockdown or ZEB1 knockdown in the ODG-treated cells. The viability of the OGD-treated cells overexpressing FOXO3a was relatively elevated by SPRY2 knockdown (Fig. 7b). Western blot analysis revealed that SPRY2 knockdown or ZEB1 knockdown caused a reduction in the protein expression of Bax and cleaved caspase 3 and an increase in Bcl-2 protein expression in the OGD-treated cells. The expression patterns of the aforementioned proapoptotic and antiapoptotic proteins in the OGD-treated cells mediated by oe-FOXO3a were reversed by sh-SPRY2 (Fig. 7c). Overall, the above results demonstrated that FOXO3a deteriorated hypoxic neuronal cell injury in the hippocampus by augmenting SPRY2 transcription.

### MeCP2 overexpression retards ischemic brain injury in vivo by disrupting FOXO3a/SPRY2 signaling

**Fig. 8:**
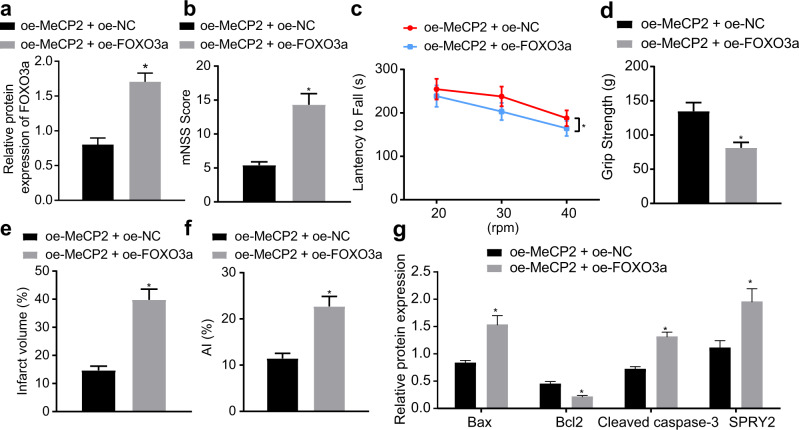
MeCP2 protects mice against ischemic brain injury *via* the FOXO3a/SPRY2 signaling axis. **a** The expression of FOXO3a in hippocampal tissues characterized by western blot analysis; **b** The mNSS of the oe-MeCP2 + oe-FOXO3a-treated mice (*N* = 10); **c** The latency period to fall from the rod of the oe-MeCP2 + oe-FOXO3a-treated mice (*N* = 10); **d** Grip strength test for evaluating neuroethology of the oe-MeCP2 + oe-FOXO3a-treated mice (*N* = 10); **e** Infarct volume in the oe-MeCP2 + oe-FOXO3a-treated mice (*N* = 10) was measured by TTC staining; **f** Cell apoptosis rate in the hippocampus of the oe-MeCP2 + oe-FOXO3a-treated mice (*N* = 10) was characterized by TUNEL staining; **g** The expression levels of apoptotic proteins (Bax, Bcl2, and cleaved caspase-3) and the expression of SPRY2 evaluated by western blot analysis. In Panels **a**, **b**, and **d**–**f**, the data were compared by unpaired t tests. In Panel **c**, data were compared by two-way ANOVA with a Bonferroni post hoc test. *p < 0.05 vs. the oe-MeCP2 + oe-NC-treated mice.

To elucidate the effect of MeCP2 on ischemic brain injury in mice through FOXO3a/SPRY2 signaling, we injected lentiviruses expressing oe-MeCP2 + oe-NC and oe-MeCP2 + oe-FOXO3a into the MCAO-treated mice. The results revealed that the expression of FOXO3a was increased in the hippocampus of the MCAO-operated mice injected with lentiviruses expressing oe-MeCP2 + oe-FOXO3a relative to that of the MCAO-operated mice injected with lentiviruses expressing oe-MeCP2 + oe-NC (Fig. 8a). The neurobehavioral test results indicated that the mNSS value was increased in the oe-MeCP2 + oe-FOXO3a-treated mice compared with the oe-MeCP2 + oe-NC-treated mice (Fig. 8b), while the latency to fall from the rod (Fig. 8c) and grip strength (Fig. 8d) were decreased by FOXO3a overexpression in the MCAO-treated mice in the presence of MeCP2. TTC staining demonstrated that the infarct volume reduced by MeCP2 was increased by restoration of FOXO3a in the MCAO-treated mice (Fig. 8e). TUNEL staining showed an elevated apoptosis rate after FOXO3a overexpression in the MCAO-treated mice treated with oe-MeCP2 (Fig. 8f), accompanied by increased protein expression of Bax and cleaved caspase 3 and reduced protein expression of Bcl-2 (Fig. 8g). In addition, the protein expression of SPRY2 and ZEB1 was increased by FOXO3a overexpression in the MCAO-treated mice treated with oe-MeCP2 (Fig. 8g). In conclusion, the results obtained in the aforementioned experiments showed that MeCP2 could prevent ischemic brain injury *via* blockade of the FOXO3a/SPRY2 signaling axis.

## Discussion

Ischemic brain injury can be initiated by various factors and, in many cases, can lead to long-term neurological disability and disease, consequently having a significant impact on quality of life and potentially inducing psychiatric comorbidities^2^. Recently, the incidence of ischemic stroke, which remains the leading cause of ischemic brain injury, has increased^22^. Reperfusion therapy (reperfusion with intravenous recombinant tissue plasminogen activator) is a crucial treatment for ischemic stroke; however, it needs to be utilized within 3 h of ischemic stroke and remains the only effective therapy for ischemic stroke^23^. Thus, deeper exploration of the molecular mechanisms associated with ischemic brain injury is important and is an essential foundation for the development of new and more effective clinical and preclinical treatments for this disease^24^. Extensive studies have shown that noncoding RNA^6^, miRNA^7^ or phytoestrogen^8^ can exert protective effects on neuronal tissues to prevent ischemic brain injury.

Previous studies not only revealed the underlying mechanism and involved pathways but also provided insight into the future directions of our research. MeCP2 is essential for normal brain function, and its abnormal expression leads to neuronal dysfunction, thus initiating severe neurodevelopmental disease due to its impact on chromatin structure^25^. Our study revealed reduced MeCP2 in mice with ischemic brain injury. MeCP2, as a specific DNA-binding protein, is highly enriched in neurons^9^. Given that the expression of MeCP2 was suppressed in ischemic neuronal injury, we hypothesized that overexpression of MeCP2 may protect mice from ischemic brain injury. Data have shown that elevation of VEGF induces the accumulation of phospho-serine 292 (pS292) MeCP2 in reactive astrocytes in the adult rat striatum following cerebral ischemia, which was accompanied by a substantial increase in reactive astrocytes^10^. Deficiency of MeCP2 expression has been identified as a cause of Rett syndrome^26,27^. Furthermore, decreased expression of MeCP2 following prenatal exposure to alcohol during pregnancy in the prefrontal cortex and striatum was shown to contribute to the hyperactive, inattentive and impulsive behaviors in rodent offspring^28^. Overall, these findings presented the protective effect of MeCP2 gene overexpression on neuronal injury, but the clinical relevance warrants further investigation.

In addition, it has been reported that MeCP2 is recruited to the promoter region of FOXO3a to methylate its promoter and repress the transcription of FOXO3a, which affects cell function and autophagy^13^. FOXO3a expression was found to be upregulated in OGD-induced neurons under hypoxia-ischemia conditions^29^. In addition, silencing of the FOXO3a/Bim axis has been proposed to inhibit neuronal cell apoptosis in response to hypoxic/ischemic brain injury^30^. However, FOXO3a is involved in different pathways to protect neurons against ischemic injury; for instance, decreased FOXO3a expression is associated with attenuated neuronal apoptosis and limited brain infarct volume in hypoxia-ischemia-induced brain injury^14^. Additionally, the deletion of FOXO3a expression induced by saponins from *Aralia taibaiensis* was shown to protect neurons from ischemia/reperfusion-induced apoptosis and delay the resultant brain cell injury^31^. Thus, regarding the unique role played by FOXO3a in the regulation of ischemic brain injury, we believe that MeCP2 confers protection to neuronal cells against ischemic injury by suppressing the expression of FOXO3a.

Further mechanistic investigation revealed that FOXO3a accelerated SPRY2 expression by binding to the promoter region of SPRY2. Existing research has reported that SPROUTY impedes cell proliferation and differentiation induced by growth factors but cannot facilitate apoptosis^32^. Given that SPRY2 was discovered as a target gene of FOXO3a^17^ while the silencing of SPRY2 by siRNA can alleviate ischemic brain injury^18^, we reasoned that inhibition of FOXO3a might attenuate ischemic brain injury by regulating the transcription of its downstream target gene SPRY2. The results of our in vivo assay also confirmed that MeCP2 overexpression could protect mice from ischemic brain injury. Finally, our data revealed that MeCP2 limited FOXO3a expression, which subsequently suppressed transcription of the downstream target gene SPRY2 and finally led to a reduction in the infarct volume and neuronal cell apoptosis. Altogether, based on our data presented here, we concluded that MeCP2 could prevent ischemic brain injury by inactivating the FOXO3a/SPRY2 signaling axis.

**Fig. 9:**
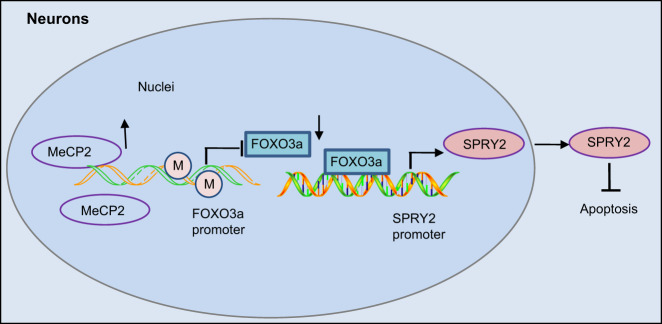
Schematic diagram of the mechanism by which MeCP2 affects ischemic brain injury. MeCP2 can promote the methylation of the FOXO3a promoter and then inhibit its expression, leading to the suppression of SPRY2 transcription and expression and thus inhibiting the apoptosis of hippocampal neurons and protecting mice from ischemic brain injury.

The molecular mechanism of how MeCP2 protected mice against ischemic neuronal injury was identified during the current study, with our data demonstrating that the overexpression of MeCP2 could facilitate promoter methylation of FOXO3a and then downregulate its expression, ultimately diminishing the enrichment of FOXO3a in the SPRY2 promoter region, which in turn inhibited the transcription of SPRY2 (Fig. 9). In conclusion, the key findings of our study suggest that MeCP2 could potentially protect against ischemic brain injury through inhibition of the FOXO3a/SPRY2/ZEB1 signaling axis, which provides new mechanistic insights for an understanding of the role of MeCP2 in ischemic neuronal injury and lays the groundwork for more effective therapeutic strategies for this disorder.

## Supplementary information

Supplementary Information
