# Supplementary material for: MeCP2 inhibits ischemic neuronal injury by enhancing methylation of the FOXO3a promoter to repress the SPRY2-ZEB1 axis
Source: Exp Mol Med. 2022 Aug 1;54(8):1076–85. doi: 10.1038/s12276-022-00790-4 (PMC9440071; doi:10.1038/s12276-022-00790-4)
Supplement: Supplementary file 1 — Supplementary Information [file 12276_2022_790_MOESM1_ESM.pdf]

# Supplementary Information

**Supplementary Fig. 1. a**. Representative protein bands of Fig. 1f. **b**. Representative protein bands of Fig. 2b. **c**. Representative protein bands of Fig. 4a. **d**. Representative protein bands of Fig. 4h. **e**. Representative protein bands of Fig. 6b. **f**. Representative protein bands of Fig. 6g.

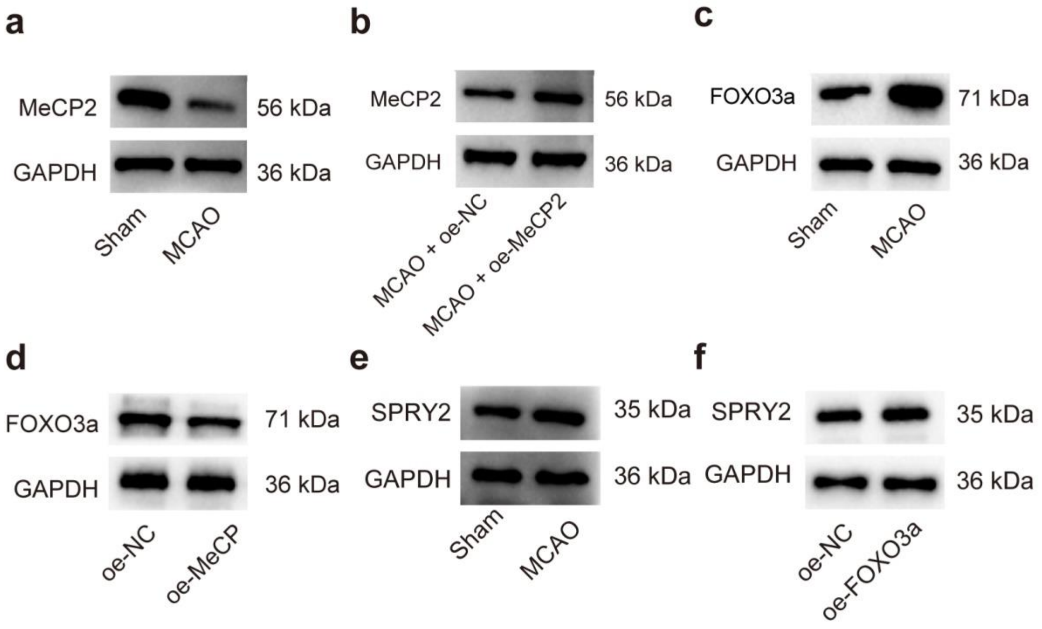

**Supplementary Table 1.** Primer sequences used for RT-qPCR

| Gene | Primer sequence |
|---|---|
| MeCP2 | Forward: 5’-AGTCTTCCATACGGTCTGTGCA-3’<br>Reverse: 5’-CTTCCCGCTTTTCTCACCAAG-3’ |
| FOXO3a | Forward: 5’-TGCTGACGGGTTGGATTT-3’<br>Reverse: 5’-CCAGTGAAGTTCCCCACATT-3’ |
| SPRY2 | Forward: 5’-ATTCAAGGGAGAGGGGTTGG-3’<br>Reverse: 5’-TCCATCAGGTCTTGGCAGTG-3’ |
| GAPDH | Forward: 5’-CCTCGTCCCGTAGACAAAATG-3’<br>Reverse: 5’-TGAGGTCAATGAAGGGGTCGT-3’ |

**Supplementary Table 2.** FOXO3a methylation-specific PCR and bisulfite sequencing PCR primer sequences

| Gene | Primer sequence |
|---|---|
| FOXO3a (M) | Forward: 5’-ATATACGTGTGTTGGTAAATAAGCG-3’ |
| | Reverse: 5’-CTAACGAAACGAAAAAAACGAA-3’ |
| FOXO3a (U) | Forward: 5’-AATAATATATATGTGTGTTGGTAAATAAGT-3’ |
| | Reverse: 5’-ACCCTAACAAAACAAAAAAAACAAA-3’ |
| Bisulfite sequencing PCR | Forward: 5’-TTTTTTTTGTTTTTGTAATTTTATT-3’ |
| | Reverse: 5’-CAAACAAATAATATCATTATCTCTACAC-3’ |

U, unmethylated; M, methylated.
